# Effect of *Bifidobacterium* upon *Clostridium difficile* Growth and Toxicity When Co-cultured in Different Prebiotic Substrates

**DOI:** 10.3389/fmicb.2016.00738

**Published:** 2016-05-18

**Authors:** L. Valdés-Varela, Ana M. Hernández-Barranco, Patricia Ruas-Madiedo, Miguel Gueimonde

**Affiliations:** Microbiology and Biochemistry of Dairy Products, Probiotics and Prebiotics, Instituto de Productos Lácteos de Asturias–Consejo Superior de Investigaciones CientíficasVillaviciosa, Spain

**Keywords:** probiotics, prebiotics, inhibition, *Clostridium difficile*, *Bifidobacterium*, toxin, HT29, RTCA

## Abstract

The intestinal overgrowth of *Clostridium difficile*, often after disturbance of the gut microbiota by antibiotic treatment, leads to *C. difficile* infection (CDI) which manifestation ranges from mild diarrhea to life-threatening conditions. The increasing CDI incidence, not only in compromised subjects but also in traditionally considered low-risk populations, together with the frequent relapses of the disease, has attracted the interest for prevention/therapeutic options. Among these, probiotics, prebiotics, or synbiotics constitute a promising approach. In this study we determined the potential of selected *Bifidobacterium* strains for the inhibition of *C. difficile* growth and toxicity in different carbon sources. We conducted co-cultures of the toxigenic strain *C. difficile* LMG21717 with four *Bifidobacterium* strains (*Bifidobacterium longum* IPLA20022, *Bifidobacterium breve* IPLA20006, *Bifidobacterium bifidum* IPLA20015, and *Bifidobacterium animalis* subsp. *lactis* Bb12) in the presence of various prebiotic substrates (Inulin, Synergy, and Actilight) or glucose, and compared the results with those obtained for the corresponding mono-cultures. *C. difficile* and bifidobacteria levels were quantified by qPCR; the pH and the production of short chain fatty acids was also determined. Moreover, supernatants of the cultures were collected to evaluate their toxicity using a recently developed model. Results showed that co-culture with *B. longum* IPLA20022 and *B. breve* IPLA20006 in the presence of short-chain fructooligosaccharides, but not of Inulin, as carbon source significantly reduced the growth of the pathogen. With the sole exception of *B. animalis* Bb12, whose growth was enhanced, the presence of *C. difficile* did not show major effects upon the growth of the bifidobacteria. In accordance with the growth data, *B. longum* and *B. breve* were the strains showing higher reduction in the toxicity of the co-culture supernatants.

## Introduction

*Clostridium difficile* is often present in the intestinal microbiota of both infants and adults, where it may be found in about 70 and 17% of the subjects, respectively (Ozaki et al., 2004; Jangi and Lamont, 2010). However, this microorganism is also the main causative agent of antibiotic associated diarrhea in nosocomial environments (Leffler and Lamont, 2015). The epidemiology of *C. difficile* infection (CDI) is changing, with an increasing occurrence in populations traditionally considered of low-risk (Carter et al., 2012), likely due to the appearance of hipervirulent strains (Rupnik et al., 2009; Yakob et al., 2015). CDI is treated with antibiotics but a high rate of recurrence is present. In this context, new therapeutic alternatives for treating or preventing CDI are being continuously explored, among them the inhibition of *C. difficile* growth by the use of probiotics or prebiotics has been tested (Ambalam et al., 2015; Auclair et al., 2015; Forssten et al., 2015).

In general, probiotics and prebiotics have been proposed as biotherapeutic agents to prevent the dysbiosis caused by antibiotics or infections, and to help the microbiota restoration after it (Reid et al., 2011). The development of food products targeting at the inhibition of *C. difficile* constitutes an interesting approach in the context of the marketing of products bearing health claims. Reducing the intestinal levels of specific pathogens, such as *C. difficile*, has been considered by the European Food Safety Authority (EFSA) as a beneficial physiological effect [(EFSA Panel on Dietetic Products, Nutrition and Allergies (NDA), 2011)]. Therefore, such an effect would constitute an opportunity for the development of food products bearing a health claim in the area of gastrointestinal health.

To date, different probiotic strains and prebiotic substrates have been reported to increase colonization resistance against *C. difficile* (Hopkins and Macfarlane, 2003; Kondepudi et al., 2014; Auclair et al., 2015; Forssten et al., 2015). In addition to their microbiota-modulatory properties, probiotics have been found to protect against infections by other mechanisms, such as production of antimicrobial compounds or competition by adhesion sites or nutrients (Servin, 2004). The ability of certain probiotics, mainly bifidobacteria and lactobacilli, to inhibit *in vitro* the adhesion of *C. difficile* to intestinal epithelial cells or intestinal mucus is well established (Collado et al., 2005; Banerjee et al., 2009). Similarly, the ability to produce antimicrobials inhibiting the growth of *C. difficile in vitro* has been repeatedly reported (Lee et al., 2013; Schoster et al., 2013). However, other potential targets of probiotics and prebiotics on CDI, such as their impact on toxin production by the pathogen, and/or toxin activity, have been explored to a lesser extent and have not attracted attention until recently (Kondepudi et al., 2014; Yun et al., 2014; Andersen et al., 2015). Ambalam et al. (2015) recently reported the ability of cell-free supernatants from some *Lactobacillus* strains, and a probiotic mix, to inhibit the growth of *C. difficile* strains in variable way depending on the carbon source used. Moreover, the authors observed a reduction of toxin titers in those *C. difficile* cultures with inhibitory cell-free supernatants added. Moreover, we have demonstrated that incubation of toxigenic *C. difficile* cell-free culture supernatants with specific bifidobacterial strains reduces the cytotoxic effect upon human epithelial intestinal cells (Valdés et al., 2016). However, the influence of prebiotic substrates upon *C. difficile* growth and toxicity when co-cultured with bifidobacteria remains largely unknown.

In this context the aim of this study was to evaluate *in vitro* the potential of four bifidobacterial strains for inhibiting the growth of *C. difficile* when co-cultured with different prebiotics as carbon source. Moreover, the effect of the strains and prebiotics on the toxicity of the co-culture supernatants upon human intestinal epithelial cells (HT29) was also determined.

## Materials and methods

### Bacterial strains and culture conditions

The widely used probiotic strain *Bifidobacterium animalis* subsp. *lactis* Bb12 and three strains of bifidobacteria from IPLA culture collection, two of them isolated from infant's feces (*Bifidobacterium longum* IPLA20022 and *Bifidobacterium bifidum* IPLA20015) (Solís et al., 2010) and the other one from breast-milk (*Bifidobacterium breve* IPLA20006) (Arboleya et al., 2011), were used. These last three strains were selected based on the good ability to reduce toxicity of *C. difficile* supernatants (Valdés et al., 2016). With regard to *C. difficile* we used the strain LMG21717, known to produce TcdA toxin and also, although at lower quantities, TcdB. This strain belongs to ribotype 001, which is one of the most common ones found in Europe (Martin et al., 2016). The *Bifidobacterium* strains were routinely grown in MRS (Biokar Diagnostics, Beauvois, France) supplemented with 0.25% L-cysteine (Sigma-Chemical Co., St. Louis, MO, USA) in an anaerobic chamber MG500 (Don Whitley Scientific, Yorkshire, UK) and *C. difficile* was grown in Reinforced Clostridial Medium (RCM, Oxoid, Thermo Fisher Scientific Inc., Waltham, MA) in Hungate tubes as previously described (Valdés et al., 2016). Overnight cultures (18 h) of the bifidobacterial strains and 13 h-old cultures of *C. difficile* were used to inoculate the batch culture fermentations.

For the batch mono- and co-culture fermentations a defined medium with the following composition was used: proteose peptone (10 g/L) (BD-Difco, New Jersey, EE.UU.), beef extract (10 g/L) (BD-Difco), yeast extract (5 g/L) (BD-Difco), polysorbate 80 (1 mL/L) (Sigma), ammonium citrate (2 g/L) (Sigma), sodium acetate (5 g/mL) (Sigma), magnesium sulfate (0.2 g/L) (Probus, Barcelona, Spain), manganese sulfate (0.056 g/L) (Panreac, Barcelona, Spain), and dipotassium phosphate (2 g/L) (Merck, New Jersey, EE.UU). Pairwise combinations of the *C. difficile* strain with the different *Bifidobacterium* strains, as well as the corresponding monocultures, were performed in the medium described above with a 2% (w/v) of different commercial prebiotic substrates added [Synergy 1 (Beneo-Orafti, Barcelona, Spain), Inulin (Sigma) and Actilight (Beghin Meiji and Tereos Syral, Marckolsheim, France)], glucose or without adding any carbon source (used as control). Each media was distributed into Hungate tubes which were inoculated with different *Bifidobacterium* strains at a final level of about 10^5^ CFU/ml in case of *B. longum*/*B. breve* and 10^4^ CFU/ml in case of *B. bifidum*/*B. animalis*, with *C. difficile* strain at final level of 10^6^ CFU/ml or with both of them, in the case of the co-culture. The bifidobacteria were inoculated at a different level depending on the strain with the aim of allowing a balanced growth of both microorganisms (bifidobacteria and clostridia). The appropriate inoculum size was determined in previous experiments (data not shown).

Co-cultures, and the corresponding mono-cultures, in different carbon sources were carried out in triplicate under anaerobic conditions at 37°C for 24 h. Samples were taken at 0 and 24 h for bacterial growth assessment by quantitative PCR (qPCR), quantification of SCFA by Gas Chromatography (GC), pH measurements (pH meter Basic 20+, Crison Instruments S.A., Barcelona, Spain), and toxigenicity determination. One milliliter of each mono-culture or co-culture was centrifuged (16,000 × g for 10 min), and pellets and supernatants were collected. For toxigenic activity upon HT29 cells, the pH of 0.7 ml cell-free supernatant from each batch culture was adjusted to 7.55 ± 0.05 with 1 and 0.1 N NaOH. All supernatants and pellets were immediately frozen at −80°C until use.

### Quantification of bacterial growth by qPCR

DNA was extracted from pellets of batch cultures using the GenElute Bacterial Genomic DNA Kit (Sigma) and kept at −80°C until analyzed. The levels of *C. difficile* and bifidobacteria in the cultures were determined as DNA copies per ml by qPCR using previously described primers and conditions (Arboleya et al., 2012). Reactions were performed on MicroAmp optical plates sealed with MicroAmp optical caps (Applied Biosystems, Foster City, CA, USA) with a 7500 Fast Real-Time PCR System (Applied Biosystems) using SYBR Green PCR Master Mix (Applied Biosystems). One microlitre of template DNA was used in the 25 mL PCR mixture. Standard curves were made with pure cultures of *B. longum* NCIMB8809 and *C. difficile* LMG 21717. In all cultures the levels of the microorganisms were above the corresponding detection limit of the technique (1 × 10^3^ and 3 × 10^3^ for bifidobacteria and *C. difficile*, respectively). Samples were analyzed by duplicate in at least two independent PCR runs.

### Determination of the production of short chain fatty acids by GC-MS

Cell-free supernatant (0.1 mL) from each batch culture was mixed with 1 ml methanol, 0.1 ml internal standard solution (2-ethylbutyric 1.05 mg/ml), and 0.1 ml 20% formic acid. This mixture was centrifuged and the supernatant obtained was used for quantification of SCFA by GC in a system composed of a 6890NGC injection module (Agilent Technologies Inc., Palo Alto, Ca, USA) connected to a flame injection detector (FID) and a mass spectrometry (MS) 5973N detector (Agilent) as described previously (Salazar et al., 2011).

### Monitoring the cytotoxic effect of the culture supernatants upon intestinal epithelial cells

The intestinal cell line HT29 (ECACC 91072201) was purchased from the “European Collection of Cell Cultures” (Salisbury, UK) and stored under liquid N_2_. McCoy's Medium (MM) supplemented with 10% fetal serum bovine, 3 mM L-glutamine and a mixture of antibiotics (50 μg/ml streptomycin-penicillin, 50 μg/ml gentamicin, and 1.25 μg/ml amphotericin B) was used for HT29 cultivation. All media and reagents were purchased from Sigma-Aldrich. Maintenance of the cell line, between passages 145 and 149, was performed under standard conditions at 37°C 5% CO_2_ atmosphere, in a CO_2_-Series Shel-Lab incubator (Sheldon Manufacturing Inc., OR, USA). The experimental procedures were carried out with the cell passage 149.

We used an RTCA (real time cell analyser) xCelligence (ACEA Bioscience Inc., San Diego, CA) system, introduced in a Heracell-240 Incubator (Thermo Electron LDD GmbH, Langenselbold, Germany) set at 37°C with 5% CO_2_ atmosphere, to monitor HT29 cells behavior. A method previously described, allowing the assessment of the damage caused by *C. difficile* supernatants, was used (Valdés et al., 2015). This method is based in the real-time monitoring of the cell index (CI). This CI is an arbitrary unit that measures the impedance, in gold-microelectrodes coating the surface of E-plates, which changes as consequence of the HT29 cells attachment and growth.

In short, 16-well E-plates were seed with 2 × 10^5^ HT29 cells (in 100 μl), hold in the RTCA equipment, incubated for 22 h to ensure the formation of a monolayer (confluent state) and the CI was monitored (recording signal every 15 min). After this incubation the medium was removed from the wells and the methodology followed was slightly different depending on the experiment. To determine the effect of the carbon source on the toxicity of *C. difficile*, 200 μL of MM containing different concentrations (from 0.63 to 40%, v/v) of cell-free neutralized-supernatants from *C. difficile* mono-cultures were added to the wells. EC50 values (concentration at which half of the maximum damage was detected) for the cultures, in the different carbon sources tested, were then calculated as previously described (Valdés et al., 2015). To determine the effect of bifidobacteria on the toxigenic capability of *C. difficile* in the different carbon sources, 200 μL of MM containing a 5% (v/v) of the neutralized supernatant from each mono- and co-culture were added to the wells. Additionally, wells filled with 200 μl of MM (non-cytotoxic control) were included in each experiment. Then, monitoring continued (recording signal every 10 min) up to 20 h under standard incubation conditions. The data analyses were carried out through RTCA software 1.2.1 (ACEA Bioscience). The CI values were normalized as previously described (Valdés et al., 2015) by dividing the CI at every point by the CI at time zero (the time of the supernatant addition, thus making the CI equal to 1 at this time) and then referred to the normalized CI of the control sample (MM) (the normalized-CI of the control sample is then the “0 line” shown in figures).

Toxin A concentration in the supernatant of *C. difficile* mono-cultures in different carbon sources was determined by ELISA test (tgcBIOMICS GmbH, Bingen, Germany).

### Statistical analysis

To asses differences among carbon sources or between mono- and co-cultures, one-way ANOVAs followed by SNK (Student-Newman-Keuls, *p* < 0.05) mean comparison test were performed. The statistical package IBM SPSS Statistics for Window Version 22.0 (IBM Corp., Armonk NY) was used to carry out these analyses.

## Results and discussion

### Inhibition of *C. difficile* growth when co-cultured with *Bifidobacterium* strains in different carbon sources

There is a great scientific interest on the development of interventions for preventing or treating CDI, including vaccines (Senoh et al., 2015), antimicrobials (Gebhart et al., 2015; Vickers et al., 2015), anti-toxin antibodies (Yang et al., 2015), or genetically engineered bacteria producing them (Andersen et al., 2015), among others. Fecal transplants have demonstrated a high efficacy to treat recurrent CDI (Lee et al., 2016), underlining the importance of the gut microbiota in this disease. Probiotics and prebiotics constitute another interesting option although differences among strains and substrates seem to exist (Allen et al., 2013).

**Figure 1:**
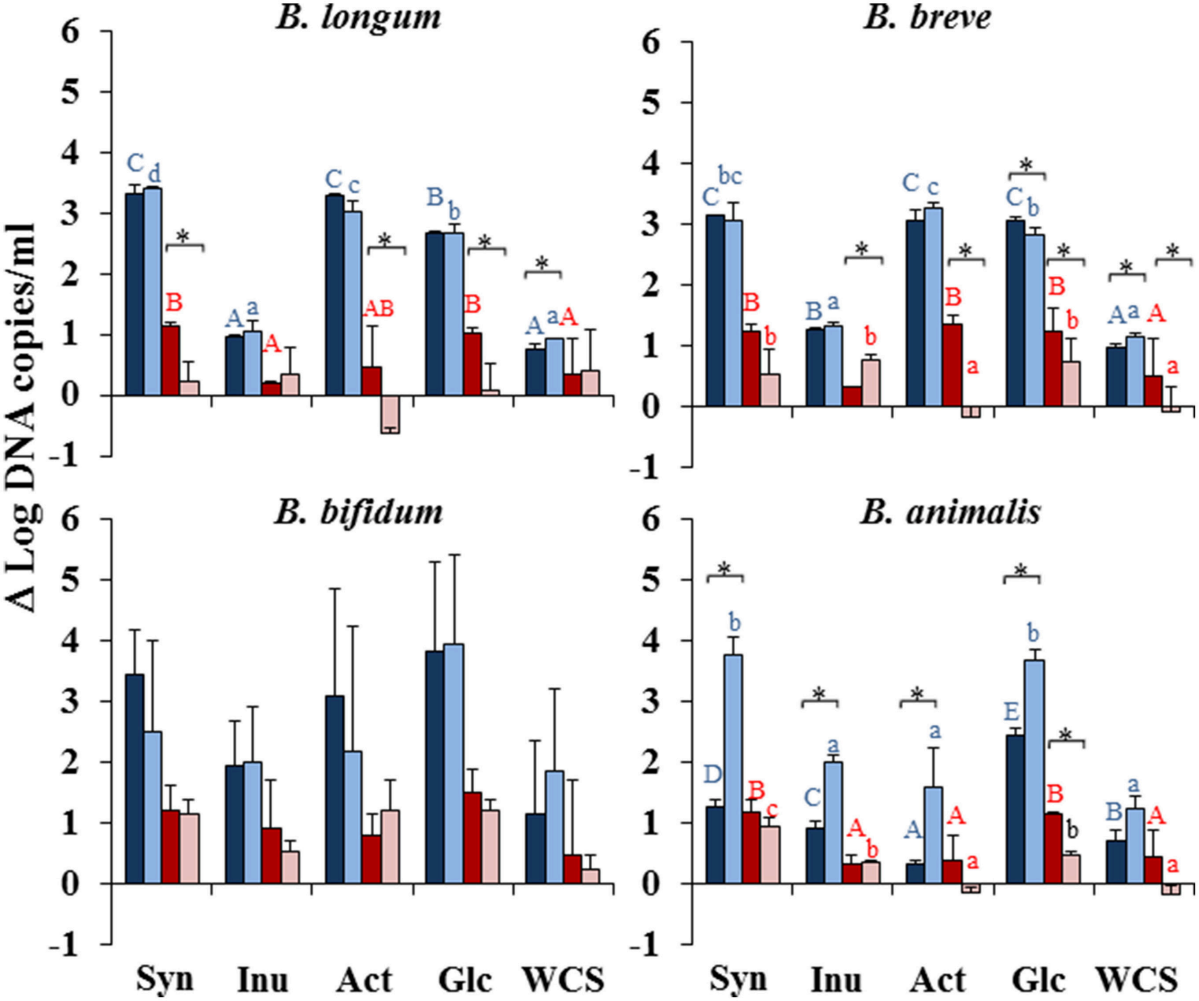
**Increments, with respect to time zero, on the levels (Log CFU/mL) of the strains when grown in mono-culture (***Bifidobacterium*** dark-blue column and ***C. difficile*** dark-red) or co-culture (***Bifidobacterium*** light-blue and ***C. difficile*** light-red column) in the prebiotics Synergy (Syn), Inulin (Inu), and Actilight (Act), in glucose (Glc) or without any carbon source added (WCS)**. Different capital letters above columns denote statistically significant differences (*p* < 0.05) among carbon sources in the mono-cultures of the corresponding bacterial strain, whereas different lowercase letters indicate differences in the co-cultures (either Bifidobacterium in blue letters or *C. difficile* in red letters). ^*^Indicates statistically significant differences (*p* < 0.05) for the corresponding bacterial strain between mono- and co-culture within the same substrate.

**Figure 2:**
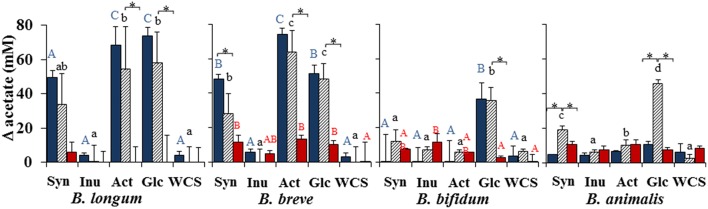
**Increments, with respect to time zero, in the concentration of acetate on the bacterial cultures when grown in mono-culture (***Bifidobacterium*** blue-bars and ***C. difficile*** red-bars) or in co-culture (stripped bars) in the prebiotics Synergy (Syn), Inulin (Inu), and Actilight (Act), in glucose (Glc) or without any carbon source added (WCS)**. Different letters above columns denote statistically significant differences (*p* < 0.05) among carbon sources in the corresponding bacterial cultures, either mono-cultures (capital letter; red color for bifidobacteria and blue color for *C. difficile*) or co-cultures (lowercase letters). ^*^Indicates statistically significant differences (*p* < 0.05) for the corresponding bacterial strain between mono- and co-culture.

In our study the mono-culture of the *Bifidobacterium* strains (Figure 1) in different substrates (dark colored bars) showed that all the strains grew well in glucose. In agreement with previous reports (Rossi et al., 2005; Kondepudi et al., 2012), the strains showed the ability to grow in short-chain fructooligosaccharides (Synergy and Actilight) (scFOS) but they were not able to grow, or did it poorly, in Inulin (Figure 1). This observation was further supported by the production of bacterial metabolites (Figure 2, Supplementary File) and the pH (Supplementary Figure 1), which in the case of Inulin remained similar to those of the negative control without carbon source added (WCS). Interestingly, *B. longum* IPLA20022 showed a significantly higher growth (*p* < 0.05) in the prebiotics Synergy and Actilight than in glucose (Figure 1), whereas no statistically significant differences were observed for *B. breve* IPLA20006 or *B. bifidum* IPLA20015 between glucose and these two prebiotics. The mono-cultures of *B. animalis* Bb12 showed a significantly lower (*p* < 0.05) growth in all prebiotics than in glucose. This strain exhibited the lowest growth of all bifidobacteria in glucose, Synergy, and Actilight (Figure 1), which correlates with the limited drop in pH observed for this strain after 24 h of incubation (Supplementary Figure 1). With regard to the pathogen, *C. difficile* grew well in Synergy, not differing significantly from glucose, and to a lower extent in Actilight (Figure 1). Therefore, in spite of generally claimed high specific fermentation of prebiotic substrates, some intestinal pathogens may also be able to ferment and grow in some of them. This underlines the importance of a careful selection of the most appropriate strains, substrates, and combinations.

When co-cultured with *C. difficile* in the different carbon sources, the behavior of the bifidobacteria was, in general, similar to that observed in the mono-cultures. We observed increases in bifidobacterial counts in glucose, Synergy, and Actilight and poor grow in Inulin. Regarding *C. difficile*, it grew better in glucose, followed by Synergy, which is in agreement with the mono-culture data, but the growth in Actilight was, in general, significantly (*p* < 0.05) worse in co-culture, the contrary being true for Inulin (Figure 1). This growth behavior of *C. difficile* in the different carbon sources was further confirmed by the metabolites production pattern (Supplementary File), showing in general a lower production of *C. difficile* metabolites, such as propionate or branched-SCFA, in co-culture with Actilight as carbon source than in the corresponding mono-culture, whilst the contrary was observed for Inulin.

When co- and mono-cultures were compared within the same carbon source, the growth of *C. difficile* was significantly reduced (*p* < 0.05) by *B. longum* IPLA20022, *B. breve* IPLA20006, or *B. animalis* Bb12 in glucose. The first two microorganisms also reduced *C. difficile* growth when co-cultured in Actilight and, in the case of *B. longum* also when Synergy was used as carbon source (Figure 1). On the contrary, no statistically significant differences between mono- and co-cultures were observed for *B. bifidum* in any carbon source. These results showed a good correlation with the pattern of production of *C. difficile* metabolites and the drop in pH (Supplementary File). This suggests the production of organic acids, with the concomitant reduction of the pH, as an important mechanism of inhibition (Tejero-Sariñena et al., 2012).

These results point out at *B. longum* IPLA20022 and *B. breve* IPLA20006, and the prebiotics Synergy and Actilight, as the most promising alternatives for inhibiting the growth of *C. difficile*. Moreover, they suggest that the pathogen inhibition is strain and substrate specific, which is in agreement with previous reports (Kondepudi et al., 2012; Tejero-Sariñena et al., 2013; Ambalam et al., 2015). Interestingly, the growth of *C. difficile* was significantly increased (*p* < 0.05) by *B. breve* in the presence of Inulin, indicating a potential risk of such combination and underlining the importance of a careful strain and substrate specific assessment.

Interestingly, effects of the co-culture with *C. difficile* on the growth of the bifidobacterial strains were also observed. Whilst in glucose the co-culture with the clostridia did not affect the growth of *B. longum*, it significantly (*p* < 0.05) reduced that of *B. breve* but increased that of *B. animalis*. Moreover, the growth of the latter microorganism was also increased by the presence of *C. difficile* in the three prebiotics tested, mainly Synergy (Figure 1) which was further confirmed by an enhanced production of acetate in the co-culture than in the corresponding monoculture (Figure 2) and a higher drop in pH (Supplementary Figure 1).

### The carbon source determines the toxicity of *C. difficile* supernatants

**Figure 3:**
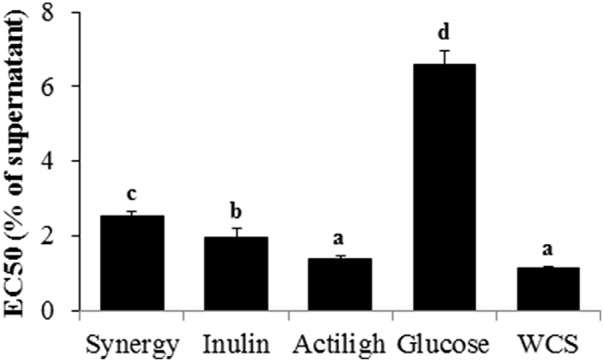
**Concentration (% v/v) of supernatants of ***C. difficile*** mono-cultures, in the different carbon sources tested, showing 50% of the maximum cell damage (EC50)**. To calculate EC50s the cell indexes obtained after 12 h of incubation of the HT29 cells with supernatants were used. Different letters above the columns denote statistically significant differences (*p* < 0.05).

In addition to bacterial growth, inhibiting the toxicity caused by *C. difficile*, for example by reducing toxin production or toxic activity, represents another target in CDI (Trejo et al., 2010, 2013). The toxicity of *C. difficile* culture supernatants has been found to be dependent on the culture media used (Valdés et al., 2015), suggesting a potential role of the carbon source available. Therefore, it is important to know whether the availability of different prebiotics as carbon source may have an impact on the toxicity of *C. difficile*. To clarify this point we determined the toxicity of neutralized cell-free supernatants, obtained from *C. difficile* monocultures after 24 h of incubation in the different carbon sources, upon the human epithelial cell line HT29 by using a real-time monitoring system (RTCA). To this end the EC50 values, defined as the concentration of supernatant causing 50% of the maximum cell damage, were calculated (Valdés et al., 2015). Supernatants obtained from the mono-cultures carried out without any carbon source or with Actilight added were significantly (*p* < 0.05) more toxic than the others (Figure 3). They showed EC50 values below 2%, which means that a concentration of monoculture supernatant lower than 2% already produced half of the maximum cell damage. On the contrary, the supernatant of the mono-culture in glucose resulted significantly (*p* < 0.05) less toxic than all the others (EC50 value over 6%), followed by that on Synergy and the one carried out with Inulin as carbon source (Figure 3). The method used (Valdés et al., 2015) allowed us to determine that the *C. difficile* supernatants' toxicity was higher when no carbon source was added or when the available carbon source supported only a limited growth of the pathogen, such as in the case of Actilight. On the contrary, the supernatant obtained when the pathogen was grown in glucose, in spite of the good growth of *C. difficile*, resulted less toxic. The availability of rapidly metabolizable sugars has been reported to inhibit toxin synthesis in *C. difficile* (Bouillaut et al., 2015). This inhibition is mediated through repression of *treR* (also known as *tdcR*), an alternative sigma factor responsible for the positive regulation of *toxA* and *toxB* genes (Mani et al., 2002). Our results seem to confirm the higher production of toxins by *C. difficile* under nutrient limitation or stress conditions in which readily fermentable sugars are not available. Moreover, in *C. difficile* a co-induction of metabolic pathways, such as that of butyrate production, and toxin production has been reported (Karlsson et al., 2000). In our study the *C. difficile* monoculture grown in glucose showed, in general, lowest butyrate production than those carried out with Synergy, Actilight or WCS added, which is in good agreement with the lower toxin production in glucose. However, the culture of the strain in Inulin, in spite of a lower production of butyrate than that in glucose, showed higher toxin concentrations, comparable to those found WCS or in the other prebiotics tested. These results indicate that, at least in some circumstances, toxin production by *C. difficile* is uncoupled from the production of metabolites such as butyrate.

**Figure 4:**
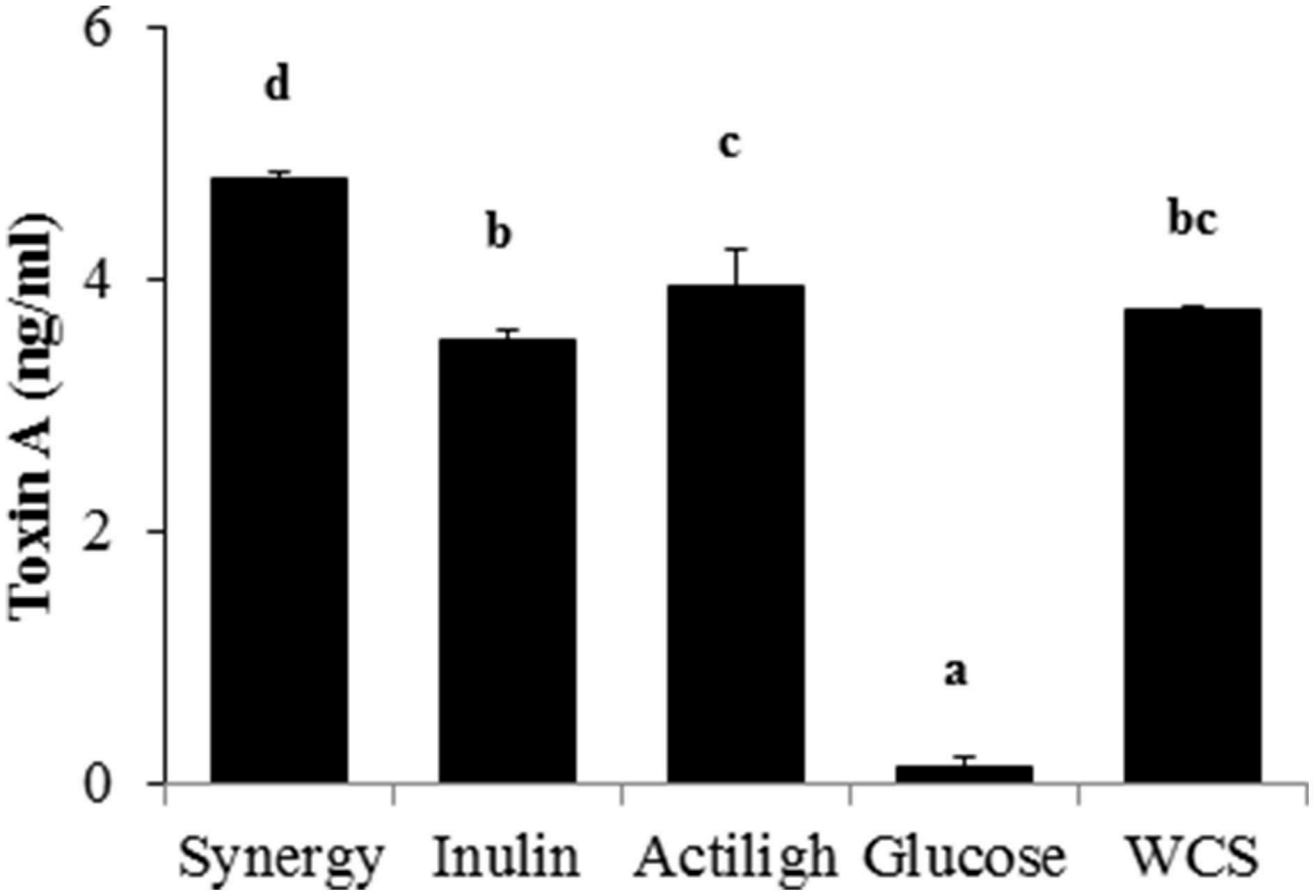
**Toxin A concentration in the different ***C. difficile*** supernatants obtained when the microorganism was growth in the different carbon sources**. Different letters above the columns denote statistically significant differences (*p* < 0.05).

In accordance with the above mentioned toxicity data, the concentration of *C. difficile* toxin A showed the lowest value in the supernatant from the culture in glucose (Figure 4). The supernatants obtained from cultures grown in Synergy, Actilight showed the highest toxin concentrations, whilst those from the growth on *C. difficile* in Inulin or WCS showed intermediate levels.

### Co-culture with bifidobacteria in different carbon sources reduces *C. difficile* toxicity

The ability of certain bifidobacterial strains, such as *B. longum* IPLA20022, to remove toxins from *C. difficile* cell-free supernatants, then diminishing their cytotoxicity, has been recently reported (Valdés et al., 2016). Now we compared the toxicity of the *Clostridium*-*Bifidobacterium* co-culture supernatants with that of the pathogen monoculture. In general we observed a significant reduction on the toxicity of the supernatants in co-culture. However, differences depending on the strain and the carbon source used were also observed, confirming the high specificity of these interactions (Trejo et al., 2010). The toxicities obtained for the co-cultures in the different carbon sources were compared by using the normalized cell index (CI) obtained after 12 h of incubation of HT29 cells with a 5% of the culture supernatants. As it was the case for the monocultures, supernatants from co-cultures carried out on the different carbon sources showed differences among them (*p* < 0.05) (Table 1). Similarly to the mono-cultures, supernatants obtained in glucose showed the lowest toxicity whilst those in Inulin, or without any carbon source added, resulted the most toxic. When the supernatants of the co-cultures with the different bifidobacteria were compared with the *C. difficile* monoculture no statistically significant differences (*p* > 0.05) were obtained in media WCS added. However, in all the carbon sources tested, either glucose or prebiotics, statistically significant differences (*p* < 0.05) were observed depending on the bifidobacterial strain used (Table 1). Co-culture in Synergy or Actilight of *C. difficile* with *B. longum* IPLA20022 or *B. breve* IPLA20006 significantly (*p* < 0.05) inhibited the toxicity of the supernatant (i.e., higher normalized CI) when compared with the mono-culture of *C. difficile*. However, *B. bifidum* IPLA20015 only was able to reduce (*p* < 0.05) the toxicity of the pathogen with Actiligh as carbon source whilst *B. animalis* Bb12 did not produce toxicity inhibition in any prebiotic. The four bifidobacteria tested were able to reduce (*p* > 0.05) the toxicity of the supernatant when co-cultured in glucose, in comparison to the *C. difficile* mono-culture, but none of them did it when the carbon source was Inulin. In the latter case, even, an increase in the toxicity was observed when the pathogen was co-incubated with *B. bifidum* (Table 1), suggesting a potential risk for such combination.

**Table 1:** **Normalized cell index (mean ± sd) obtained after 12 h of incubation of HT29 cells with the supernatants (5%) of the ***C. difficile*** mono-culture or ***C. difficile***-***Bifidobacterium*** co-cultures grown in different prebiotics, glucose or without any carbon source added (WCS)**.

**Culture**	**Normalized cell index**				
	**Carbon source**				
	**Synergy**	**Inulin**	**Actilight**	**Glucose**	**WCS**
*C. difficile*	−0.39±0.03^a,1^	−0.30±0.03^a,2^	−0.35±0.04^a,1^	−0.23±0.01^b,1^	−0.34±0.04^a^
*C. difficile*–*B. longum*	−0.06±0.04^b,2^	−0.32±0.07^a,2^	−0.13±0.05^b,3^	0.01±0.02^b,3^	−0.43±0.13^a^
*C. difficile*–*B. breve*	−0.02±0.02^d,2^	−0.32±0.03^b,2^	−0.07±0.01^c,4^	0.00±0.01^d,3^	−0.37±0.01^a^
*C. difficile*–*B. bifidum*	−0.40±0.08^b,1^	−0.56±0.02^a,1^	−0.24±0.01^c,2^	0.00±0.01^d,3^	−0.34±0.01^b^
*C. difficile*–*B. animalis*	−0.35±0.02^a,1^	−0.31±0.03^a,2^	−0.32±0.02^a,1^	−0.03±0.00^b,2^	−0.34±0.02^a^

^*^Different superscripts letters within the same row indicate statistically significant differences (p < 0.05) among carbon sources, whereas different superscript numbers within the same column denote differences (p < 0.05) among cultures.

Our results show that *B. longum* IPLA20022 and *B. breve* IPLA20006 reduced the toxicity of the co-cultures with sc-FOS as carbon source. Interestingly these two strains have previously shown the ability to remove *C. difficile* toxins from solution (Valdés et al., 2016). Although the putative mechanism behind toxin inactivation remains to be elucidated, it has been demonstrated that certain microorganisms produce compounds able to degrade *C. difficile* toxins or to reduce their toxicity (Castagliuolo et al., 1996; Banerjee et al., 2009; Carasi et al., 2012; Valdés et al., 2016). These mechanisms may be involved in the effect observed by us. However, given that in our case both microorganisms are co-incubated, the direct inhibition of the growth of the pathogen and/or an modulation of the expression of the toxin genes in *C. difficile* by the presence of bifidobacteria, similarly to that previously reported for *Lactobacillus acidophilus* (Yun et al., 2014), may also be involved. Previous studies pointed out a role of organic acids, such as lactic acid, in the inhibition of both growth and toxin production by *C. difficile* (Kolling et al., 2012; Yun et al., 2014). Therefore, the ability of bifidobacteria to produce acids, mainly acetic and lactic acids, and the pH drop caused by them may partially explain our observations. However, the role of other interactions cannot be overruled, especially since behaviors not explained by the acids, such as the increased toxicity of the co-culture *C. difficile*-*B. bifidum* in Inulin, were also observed.

## Conclusion

Co-culture with *B. longum* IPLA20022 or *B. breve* IPLA20006 in the presence of scFOS, but not of Inulin, reduces significantly the growth of *C. difficile*. Moreover, co-culture with these two strains in Synergy or Actilight reduced the toxicity of the *C. difficile* supernatants. Therefore, *B. longum* IPLA20022 and *B. breve* IPLA20006, in combination with Synergy or Actilight, are the most promising strains and compounds for the development of probiotic, prebiotic, or synbiotic products targeting at the reduction of CDI. However, future *in vitro* studies aiming at other clinically relevant *C. difficile* strains, as well as *in vivo* evaluation of the efficacy of the products, would be needed before drawing firm conclusions.

## Author contributions

MG and PR contributed with the conception, experimental design, and results interpretation of this study. LV carried out all experiments, AH performed chromatographic analyses. MG was in charge of writing the drafted manuscript. All authors performed a critical revision of the manuscript and approved the final version.

### Conflict of interest statement

The authors declare that the research was conducted in the absence of any commercial or financial relationships that could be construed as a potential conflict of interest.
