# Supplementary figures and images for: Effect of Bifidobacterium upon Clostridium difficile Growth and Toxicity When Co-cultured in Different Prebiotic Substrates
Source: Front Microbiol. 2016 May 18;7:738. doi: 10.3389/fmicb.2016.00738 (PMC4870236; doi:10.3389/fmicb.2016.00738)

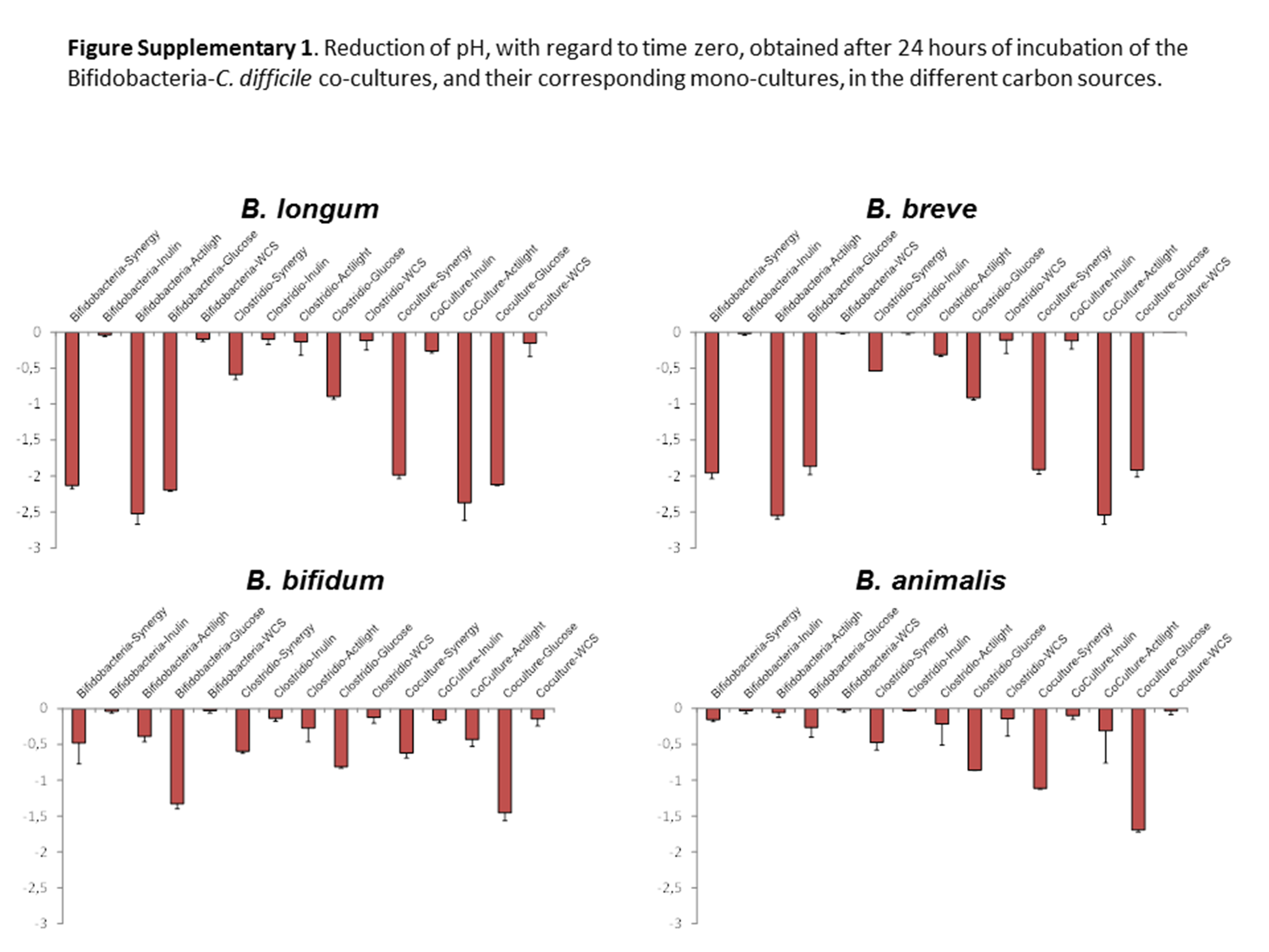

Supplement: Supplementary file 1 [file Image1.TIF]
